# Mating of escaped domestic pigs with wild boar and possibility of their offspring migration after the Fukushima Daiichi Nuclear Power Plant accident

**DOI:** 10.1038/s41598-019-47982-z

**Published:** 2019-08-08

**Authors:** Donovan Anderson, Rio Toma, Yuki Negishi, Kei Okuda, Hiroko Ishiniwa, Thomas G. Hinton, Kenji Nanba, Hidetoshi B. Tamate, Shingo Kaneko

**Affiliations:** 1 Fukushima University, Symbiotic Systems Science and Technology, Fukushima, 960-1248 Japan; 2 Hiroshima Shudo University, Faculty of Human Environmental Studies, Hiroshima, 731-3195 Japan; 3 Fukushima University, Institute of Environmental Radioactivity, Fukushima, 960-1248 Japan; 4 Yamagata University, Department of Biology, Yamagata, 990-8560 Japan

**Keywords:** Ecological genetics, Conservation biology, Invasive species, Ecological genetics, Conservation biology

## Abstract

The 2011 Tohoku earthquake drastically changed human activities in some regions of Fukushima Prefecture, Japan. The subsequent tsunami damage and radioactive pollution from the Fukushima Daiichi nuclear power plant resulted in the evacuation of humans, and abandonment of agricultural lands, allowing population expansion of wildlife into areas formally inhabited by domesticated livestock. Unintentional escape of domesticated pigs into wildlife inhabited environments also occurred. In this study, we tested the possibility of introgression between wild boar and domesticated pigs in Fukushima and neighboring prefectures. We analyzed mitochondrial DNA sequences of 338 wild boar collected from populations in the Tohoku region between 2006 and 2018. Although most boar exhibited Asian boar mitochondrial haplotypes, 18 boar, phenotypically identified as wild boar, had a European domesticated pig haplotype. Frequencies of this haplotype have remained stable since first detection in 2015. This result infers ongoing genetic pollution in wild boar populations from released domesticated pigs. In 2018, this haplotype was detected outside of evacuated areas, suggesting migration and successful adaptation. The natural and anthropocentric disasters at Fukushima gave us the rare opportunity to study introgression processes of domestic genes into populations of wild boar. The present findings suggest a need for additional genetic monitoring to document the dispersal of domestic genes within wild boar stock.

## Introduction

The 2011 Tohoku earthquake, tsunami, and nuclear accidents in Fukushima Prefecture, Japan, drastically changed human activities in areas close to the disaster sites. Humans were required to evacuate a 20-km radius around the Fukushima Daiichi Nuclear Power Plant (FDNPP) and this altered some 650 km^2^ of abandoned villages, agricultural lands, and commercial forests, which allowed for expansion of wildlife into areas formally inhabited by humans and their domesticated livestock. The removal of humans and associated rewilding of nature allows exchange of individuals from different populations, resulting in positive attributes such as increased gene flow, abundant wildlife populations, mitigation of inbreeding and enhanced viability of wildlife populations^1–3^. However, there is also the potential hybridization or introgression of escaped domesticated livestock with wildlife^4^. Research of genetic contamination through introgression of released domesticated animals has been reported in multiple mammal populations^5,6^. Genetic contamination is a threat to natural biodiversity and needs to be monitored to help prevent future genetic contamination in mammal populations^7–9^.

Wild boar (*Sus scrofa*), which are widely distributed geographically including large areas in Asia, Europe, and North Africa^10–12^, are known to reproduce with their domestic relative^10,11,13^. Due to little reproductive isolation between pig and boar, the introgression of introduced pigs into wild boar populations may alter genetic components of local wild boar populations^14^. Potential introgression occurred following the FDNPP accident when some of an estimated 31,500 domesticated pigs left behind in the evacuation zone escaped into the wild^15^. This dispersal of released pigs into nearby environments may have long term ecological consequences by altering traits like reproduction rate or immunology of current wild boar populations^14,16,17^. Thus, there is a need to monitor potential hybridization within wild boar subpopulations within and nearby the evacuated areas (henceforth called Difficult-to-Return to zone) of Tohoku, Japan.

Previous studies of the Japanese wild boar populations have reported low level gene flow, and regionally stable genetic structures^12,18,19^. However, there is a dearth of genetic composition studies for wild boar populations in the Tohoku region of Japan making it difficult to analyze the consequences of hybridization with domestic stock following the FDNPP accident. Providing regional information on the genetic dynamics of wild boar in this area is important to better understand introgression and hybridization of wildlife following large-scale disasters.

In this study, we investigated the mitochondrial DNA (mtDNA) of wild boar in Fukushima, Yamagata, and Miyagi Prefectures with the aims to: (1) study possible changes in mtDNA genetic composition of wild boar following the FDNPP disaster, and (2) determine if the frequency of escaped domestic pig mtDNA occurrence in wild boar populations is stable, increasing or declining within Fukushima’s evacuated zones or neighboring Prefectures.

## Results

Comparative analyses among 338 sequences (713-bp) were obtained in the Tohoku region, which revealed transitional substitutions at 21 nucleotide substitution sites. Nucleotide variation of Japanese wild boar and domestic pig haplotypes are provided in Table 1. Between one and eight nucleotide differences were observed among haplotypes. With all combinations of substitutions, four haplotypes, J10 (D42172), J3 (D42174), J5 (AB015085) and H1 (MK801664), were identified for wild boar populations. J10, J3, and J5 haplotypes were previously observed in other wild boar mtDNA analysis studies^18,20,21^. The frequencies of individuals observed in haplotypes J10, J3, and J5 were 319 (92%), 5 (1.4%), and 3 (1.0%), respectively. All J3 individuals were detected within evacuation zones in 2015. J5 individuals were detected outside of evacuation areas in 2009, 2016 and 2017.

**Table 1:** Four D-loop haplotypes found in the wild boar population and seven D-loop haplotypes found in domestic pig samples, based on 21 substitution sites. “n” represents total number of collected samples detected for that haplotype.

Haplotype	n	Nucleotide number																				
		160	175	182	187	196	204	209	232	292	311	330	345	353	357	374	441	503	552	611	626	708
**Asian boar**																						
J10	319	C	A	A	—	T	T	G	C	T	C	C	G	G	T	T	T	T	G	T	G	C
J5	3	C	A	A	—	T	T	G	C	T	C	C	G	A	C	T	T	C	G	C	G	C
J3	5	C	A	A	—	T	T	A	C	T	T	C	G	A	C	T	T	T	A	T	G	T
**Pigs**																						
H1	19^a^	T	A	G	C	C	C	A	T	T	C	C	A	A	C	T	C	C	A	T	A	C
P1	1	T	T	G	C	C	C	A	C	T	C	C	A	A	C	C	C	C	A	T	A	C
P2	2	T	T	G	C	C	C	A	T	T	C	T	A	A	C	C	C	C	A	T	A	C
P3	2	T	T	G	C	C	C	A	T	T	C	C	A	A	C	C	C	C	A	T	A	C
P4	2	T	T	G	C	C	C	A	T	T	C	C	A	A	C	C	C	C	G	T	G	C
P5	1	C	A	A	—	T	T	G	C	C	C	T	G	A	T	T	T	C	G	T	G	C
P6	1	C	A	A	—	T	T	G	C	T	C	T	G	A	T	T	T	C	A	T	G	C

^a^18 individuals from collected wild boar samples, and 1 individual from purchased super market samples.

**Figure 1:**
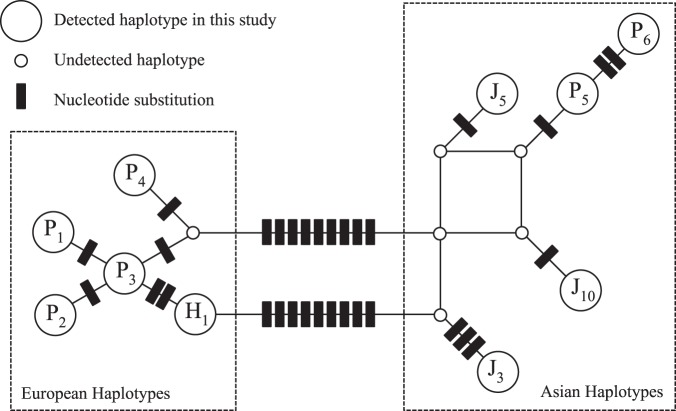
Parsimonious network constructed using 10 mtDNA haplotypes from Japanese wild boar, and European and Asian pigs. These 10 mtDNA haplotypes comprise the six European and Asian pig haplotypes and three wild boar mtDNA haplotypes (J10, J5, and J3) found in the present study.

**Figure 2:**
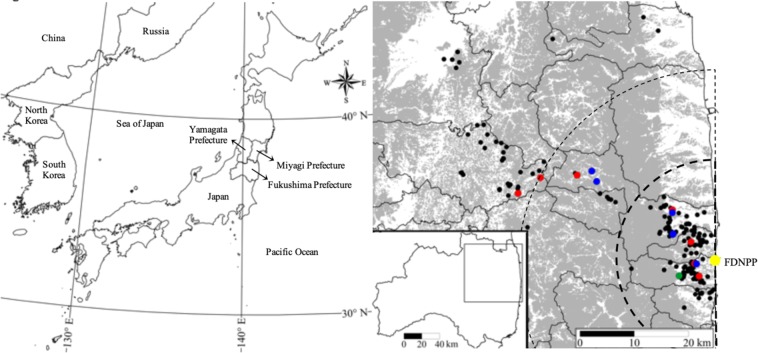
Forest distribution and sampling locations in Fukushima, Japan. The grey areas represent forest, and the dots show boar sampling locations collected from 2014–2018. A 20 and 40 km radius from the FDNPP (in yellow) are shown by dashed lines. Black dots show sample locations for wild boar haplotype (J10). Detection of H1 (domestic pig crossed with wild boar) haplotype in 2015, 2016 and 2018 are represented by green, blue, and red dots, respectively. 40 Miyagi and Yamagata Prefecture samples obtained in 2006–2011 are not shown, however GPS data are provided in supplemental information.

The observed frequency of domestic pig haplotype (H1) was 5.3% (18 individuals) amongst all samples. Haplotype network analysis suggests these 18 individuals were of European domesticated pigs, and thus H1 haplotypes were identified as a lineage that has at least one female ancestor of European domestic pigs (Fig. 1). In 2015 and 2016, all detected H1 individuals were within the initial evacuation area and Difficult-to-Return to zone of Fukushima prefecture. However, H1 individuals were detected outside the evacuation areas in 2018 (Table 2, Fig. 2).

**Table 2:** Frequency of hybrid (H1) haplotype detection before FDNPP accident and after (2014–2018) partitioned by initial evacuation zones (<20 km), Difficult-to-Return to zones (20–40 km), and non-evacuated areas (>40 km).

Distance from NPP (km)	Number of samples	H1 (%)	Year (number hybrids detected)					
			2006–2011 (before accident)	2014	2015	2016	2017	2018
<20	207	13 (6.2%)	0	0	15 (1)	52 (5)	17 (0)	123 (7)
20–40	29	2 (6.9%)	0	0	1 (0)	14 (2)	0	14 (2)
40 <	102	1 (0.1%)	46 (0)	9 (0)	14 (0)	18 (0)	4 (0)	11 (1)
All	338	18 (5.3%)	46 (0)	9 (0)	30 (1)	84 (7)	21 (0)	148 (10)

The ten pig samples taken from a slaughterhouse or local meat market in Fukushima prefecture showed seven haplotypes (P1, P2, P3, P4, P5, P6, and H1) suggesting higher genetic diversity than that of the 338 wild boar samples obtained for this study. One such pig sample’s haplotype was identical to that of H1, further suggesting that H1 boar has a domestic pig ancestor in their maternal lineage. Additionally, alignment with GenBank sequences showed two domestic pig sample sequences (P5 and P6) had mtDNA sequences similar to those found in wild boar populations (Fig. 1), suggesting that these pig samples probably have Asian boar ancestors in their maternal lineage^22,23^.

## Discussion

Genetic diversity for wild boar from this area of the Tohoku region (mainly Fukushima Prefecture, Japan) was analogous to that of wild boar in other regions of Japan^12,24^. Our mitochondrial DNA sequence data revealed a dominance of J10 haplotype in wild boar populations (91%), a result similar to what was found in Ibaraki Prefecture, Fukushima’s southern neighbor^21^, where the study’s population was 100% J10. Dominance of J10 haplotype in nearby areas suggest a strong similarity of genetic diversity and are most likely due to a stochastic effect within these areas of Japan.

Wild animal hybridization is a matter of worldwide attention^5,16,25,26^ and detection of hybridization between wild boar populations with released domestic pigs has occurred in numerous regions outside and within Japan^27,28^. Data presented here show genetic introgression from European domestic pigs into Japanese wild boar populations, which suggests the occurrence of hybridization. The 18 observed identical domestic pig mtDNA haplotypes in these individuals support a scenario of introgression from domestic pig lineages into the wild. Additionally, one mtDNA haplotype from a pig sample collected from a Fukushima slaughterhouse was identical to that found in released domesticated pigs (H1). Therefore, genetic introgression in this region could be a direct result from abandoned agriculture lands and the unintentional release of domestic pigs following the FDNPP accident. Release of domestic livestock is an inevitable consequence following a natural disaster, therefore management actions to monitor hybridization and introgression of these populations over time should be considered to understand their impact on wildlife populations.

Only a single haplotype was detected for escaped domesticated pigs and the mtDNA sequences obtained from ten pig samples showed genetic variation (Table 1), which is a result that may provide an interesting indication of the escaped domestic population and its survival in the wild. The single wild pig haplotype within our study suggests that domestic pig genetic introgression may be occurring from a single or limited source (e.g. abandoned agriculture farms within the evacuated area of Fukushima), which may have a low genetic variation among individuals. The detection of a single haplotype among escaped domestic pigs may also suggest that if multiple European and Asian pig haplotypes were released into the wild after the accident, only this haplotype lineage proved to be successful as an outcome of natural selection. Other studies of genetic variation in wild pigs generally showed mitochondrial haplotype variation^29–32^. Our findings are in contrast to these previous studies. There may be specific factors in the Fukushima area preventing other domestic pig lineages from surviving, such as a difficulty of colonization within the local environment and/or severe competition from native wild boar^9^.

Introgression and spread of released domesticated pigs can alter the genetic population structure of native wild boar^16^. Detection of H1haplotype has remained stable within the evacuation areas since 2015 (Table 2). Our first detection of the H1 haplotype outside of the evacuated areas occurred in 2018, seven years after the accident. This may indicate that the H1 lineage is spreading to other areas and presents a unique opportunity to understand continued introgression of released domestic pigs and population migration of wild boar following FDNPP. Additionally, radiocesium concentrations of some wild boar tissues have been found to be higher than regulated Japanese government limits in areas outside of evacuation zones or in areas deemed habitable to people, which also suggests boar have migrated to these areas (Anderson *et al*. unpublished data). If offspring of crossbred domesticated pigs and wild boar are spreading to other areas, continued monitoring of crossbred individuals will provide more knowledge on their migration patterns and dispersal into other regions.

Our results suggest that uncontrolled dispersal of released domestic pig populations, and consequences of drastic evironmental changes (e.g. human evacuations, loss of migratory boundaries), is leading to introgressive hybridization of local natural wild boar populations. However, mtDNA data can only infer about female lineages (i.e., a cross between a female pig and a male wild boar). Thus, our study may be underestimating the introgression or possibility of hybrid occurrences. Our findings suggest the need for microsatellite marker genetic analyses to obtain more complete information on genetic hybrids, and to better understand the consequences of escaped domesticated livestock. Additionally, future genetic studies could provide data on migration patterns and the rare opportunity to better understand invasive introgression processes of domestic gene influx into wild populations following a large-scale disaster.

## Methods

### Study area and sample collection

Muscle tissue (*Bicep femoris*) samples were collected from 338 Japanese wild boar captured in multiple municipalities within Tohoku, Japan (37° 24′N, 140° 59′E) from 2006 to 2018 (Fig. 2, Table 2). All samples had similar morphological characteristics of Japanese wild boar as described by previous studies^33^. In addition, 10 domestic pig muscle tissues were obtained from a slaughterhouse and a local meat market in Fukushima prefecture for comparison with wild boar and escaped domesticated pig sequence data. All samples were stored individually at −20 °C in 99.5% ethanol. Wild boar capture site location (GPS) and date were recorded and are provided in supplemental information. No animals were killed specifically for this research. All animals were legally culled by licensed hunters, and this entire study was approved by Fukushima University’s Institutional Animal Care and Use Committee. All experiments were performed in accordance with relevant guidelines and regulations.

### DNA extraction and mitochondrial DNA analysis

Total genomic DNA was extracted using Gentra Puregene Tissue Kit (QIAGEN), according to manufacturer’s instructions. Approximately 1 μl of extracted DNA was used to amplify the base sequence control region by polymerase chain reaction (PCR) as described by Watanobe *et al*., 2003. PCR primers, mitL3 (5′-ATATACTGGTCTTGTAAACC-3′) in L strand of threonine tRNA and mitH106 (5′-ACGTGTACGCACGTGTACGC-3′) in the H strand of the control region were used to amplify the mitDNA as described by Watanobe *et al*. 1999. PCR amplification was done in an 8 μl reaction mixture containing 2 to 30 ng of extracted DNA, 4 μL of QIAGEN Multiplex PCR Master Mix, and 0.2 μL of each 10 μM primer. Thermal cycling conditions used in T100 thermal cycler (Bio-Rad Laboratories, Inc., Hercules, CA, USA) included: initial DNA denaturation at 95 °C for 15 min, heat denaturation at 94 °C for 30 sec, annealing at 60 °C for 1 min 30 sec, extension reaction at 72 °C for 1 min. The cycle was repeated 30 times followed by the final extension at 60 °C for 30 min. The amplified DNA fragments were subjected to electrophoresis on a 1.5% agarose gel and later purified. Amplified PCR products were purified using illustra ExoStar (GE Healthcare). Sequence reaction was performed on the purified product with ABI BigDye Terminator Cycle Sequencing Kit ver. 3.1 (Applied Biosystems). As described in Watanobe *et al*. 2003, additional primers mitL120 (5′-ACCGCCATTAGATCACGAGAC-3′) and mitH124 (5′-ATGGCTGAGTCCAAGCATCC-3′) were added for DNA sequencing of the mtDNA control region. The nucleotide sequence was determined with ABI PRISM 3130 Genetic Analyzer (Applied Biosystems).

### Data analysis

The mtDNA control region of 338 samples were successfully amplified using PCR with primer pair L3/H106 or L120/H124. Using PCR product-direct sequencing with each of four internal primers (L3, L120, H106, H124), partial sequences (713-bp) were determined from all samples. DNA sequencing data was viewed from FinchTV chromatogram viewer version 1.5.0 (Geospiza Inc.). Based on aligned sequences, a parsimony network of all haplotypes was created using results from TCS 1.21^34^ with gaps treated as missing data (Fig. 1). Concurrently, all sequences were aligned with previously deposited sequences in GeneBank^12,29^ and haplotype origins (e.g. European or Japanese clade) were identified.

Due to the possible environmental impact on wild boar populations within evacuated zones following the FDNPP accident, our results of haplotype occurrence were reviewed based on samples before and after the accident (Table 2). Additionally, frequency of haplotypes was characterized by distance between sample locations and FDNPP (e.g. initial evacuation zones in 2011, Difficult-to-Return to zone, and non-evacuated areas).

### Accession codes

Detected hybrid (H1, cross between Japanese wild boar and released domestic pigs) DNA sequence has been deposited at GenBank under accession code MK801664.

## Supplementary information

GPS Location and date of samples

## Data Availability

Supplementary information (GPS data) accompanies this manuscript as a downloadable PDF file. Additional datasets generated during and/or analysed during the current study are available from the corresponding author on reasonable request.
