# Author Correction: Mating of escaped domestic pigs with wild boar and possibility of their offspring migration after the Fukushima Daiichi Nuclear Power Plant accident

**DOI:** 10.1038/s41598-019-53668-3

**Published:** 2019-11-20

**Authors:** Donovan Anderson, Rio Toma, Yuki Negishi, Kei Okuda, Hiroko Ishiniwa, Thomas G. Hinton, Kenji Nanba, Hidetoshi B. Tamate, Shingo Kaneko

**Affiliations:** 1 Fukushima University, Symbiotic Systems Science and Technology, Fukushima, 960-1248 Japan; 2 Hiroshima Shudo University, Faculty of Human Environmental Studies, Hiroshima, 731-3195 Japan; 3 Fukushima University, Institute of Environmental Radioactivity, Fukushima, 960-1248 Japan; 4 Yamagata University, Department of Biology, Yamagata, 990-8560 Japan

Correction to: *Scientific Reports* 10.1038/s41598-019-47982-z, published online 08 August 2019

This Article contains an error in Table 2 where the frequency of hybrid (H1) haplotype detection before FDNPP accident and after (2014–2018) partitioned by Difficult-to-Return zones (20–40 km) is incorrect.

The correct value should read 4(14%).

