# Supplementary material for: Mating of escaped domestic pigs with wild boar and possibility of their offspring migration after the Fukushima Daiichi Nuclear Power Plant accident
Source: Sci Rep. 2019 Aug 8;9:11537. doi: 10.1038/s41598-019-47982-z (PMC6687819; doi:10.1038/s41598-019-47982-z)
Supplement: Supplementary file 1 — GPS Location and date of samples [file 41598_2019_47982_MOESM1_ESM.pdf]

Mating of escaped domestic pigs with wild boar and possibility of their offspring migration after the Fukushima Daiichi Nuclear Power Plant accident : Supplemental file

Donovan Anderson, Rio Toma, Yuki Negishi, Kei Okuda, Hiroko Ishiniwa, Thomas G. Hinton, Kenji Nanba, Hidetoshi B. Tamate, and Shingo Kaneko

			GPS	
Boar ID	Date Sampled	Locaton	LAT	Long
150713OW	7/13/15	Okuma	37.396393	140.959649
160531-1	5/31/16	Namie	37.568932	140.778014
160531-2	5/31/16	Namie	37.500294	140.945460
160531-3	5/31/16	Namie	37.464554	140.946247
160729B-1	7/29/16	Namie	37.551871	140.788174
161101B-2	11/1/16	Namie	37.465529	140.947112
20161126-1	11/26/16	Okuma	37.416058	140.995227
20161128O-207	11/28/16	Okuma	37.416058	140.995227
180530-C1	5/30/18	Nihonmatsu	37.53161	140.62490
180702-O2	7/2/18	Futaba	37.45207	140.98398
180704-O3	7/4/18	Namie	37.56214	140.7474972
180709-O1	7/9/18	Okuma	37.39566	141.00081
181005-O2	10/5/18	Nihonmatsu	37.55762	140.67138
181027-O2	10/27/18	Okuma	37.41645	140.99158
181027-O3	10/27/18	Okuma	37.41645	140.99158
181027-O4	10/27/18	Okuma	37.41645	140.99158
181027-O5	10/27/18	Okuma	37.41645	140.99158
181128-O2	11/28/18	Namie	37.50506	140.94531
150731Na	7/31/15	Okuma	37.30115	140.98129
150814NW	8/15/15	Nihonmatsu	37.562062	140.509395
151022MW	10/22/15	Namie	37.501782	140.946236
F14	1/11/09	Fukushima	37.371407	139.32505
160617B-1	6/16/17	Fukushima	37.704008	140.401985
160617B-2	6/16/17	Fukushima	37.704008	140.401985
161215T-1	12/15/16	Tochigi	36.439514	139.642847
161215T-2	12/15/16	Tochigi	36.439514	139.642847
151119NW1	11/19/15	Namie	37.496628	140.9619
151119NW2	11/19/15	Namie	37.496628	140.9619
140515	5/14/14	Fukushima	37.749246	140.4729678
140525B	5/25/14	Fukushima	37.735239	140.498299
140526B	5/26/14	Fukushima	37.735239	140.498299
140527B	5/27/14	Fukushima	37.735239	140.498299
140607B	6/7/14	Soma	37.819648	140.8880746
140616	6/16/14	Fukushima	37.735239	140.498299
150201	2/1/15	Soma	37.7973436	140.9188975
150203SW1	2/3/15	Soma	37.819648	140.8880746
150128NB	1/28/15	Namie	37.478359	140.977474
150502SW	5/5/15	Soma	37.7973436	140.9188975
150504SW2	5/4/15	Soma	37.7973436	140.9188975
150504SW	5/4/15	Soma	37.783438	140.699192
150506SW	5/6/15	Soma	37.783438	140.699192
150510SW	5/10/15	Fukushima	37.7785	140.827384

150510SW2	5/10/15	Fukushima	37.776257	140.87701
150711NW1	7/11/15	Nihonmatsu	37.55894	140.514305
150711NW2	7/11/15	Nihonmatsu	37.55894	140.514305
150730OW	7/30/15	Okuma	37.396393	140.959649
151119NW3	11/19/15	Namie	37.470003	140.94042
151119NW4	11/19/15	Namie	37.470003	140.94042
151218NW1	12/18/15	Namie	37.506008	140.903405
151218NW2	12/18/15	Namie	37.473344	140.969423
151222NW1	12/22/15	Namie	37.491431	140.978599
151222NW2	12/22/15	Namie	37.493705	141.0005899
160126NW	1/26/16	Namie	37.493705	141.0005899
160126NW2	1/26/16	Namie	37.493705	141.0005899
160315NW	3/15/16	Namie	37.491431	140.978599
160409-1	4/9/16	NIhonmatsu	37.534089	140.589689
160409-2	4/9/16	Nihonmatsu	37.5444946	140.607215
160409-3	4/9/16	Nihonmatsu	37.5247486	140.608102
160726B-3	7/26/16	Okuma	37.4000278	140.9892444
160726B-4	7/26/16	Okuma	37.4000278	140.9892444
160728B-1	7/28/16	Katsurao	37.528640	140.798470
160731B-4	7/31/16	Namie	37.479347	140.981908
160731B-5	7/31/16	Namie	37.497798	141.014968
160603-E	6/3/16	Namie	37.523055	140.931445
160801B-1	8/1/16	Okuma	37.395130	140.973570
160801B-2	8/1/16	Tomioka	37.362790	140.996390
160801B-3	8/1/16	Fukushima	37.748730	140.490098
160916B	9/16/16	Nihonmatsu	37.566058	140.672044
160917B-2	9/17/16	Nihonmatsu	37.560993	140.658546
160917B-3	9/17/16	Nihonmatsu	37.560993	140.658546
160918B-1	9/18/16	Fukushima	37.744412	140.504675
160918B-2	9/18/16	Nihonmatsu	37.539503	140.628628
160918B-3	9/18/16	Nihonmatsu	37.536500	140.627664
160919B-1	9/19/16	Nihonmatsu	37.534920	140.589073
160919B-2	9/19/16	Nihonmatsu	37.542977	140.612194
160920B-1	9/20/16	Namie	37.465529	140.947112
160926B	9/26/16	Nihonmatsu	37.562062	140.509395
160928B	9/28/16	Nihonmatsu	37.562445	140.51106
161004B-4	10/4/16	Namie	37.480657	140.940891
161004B-5	10/4/16	Namie	37.480657	140.940891
161004B-6	10/4/16	Namie	37.480657	140.940891
161004B-7	10/4/16	Namie	37.480657	140.940891
140602SW	6/2/14	Fukushima	37.735239	140.498299
140721B-2	7/21/14	Soma	37.7973436	140.9188975
150108W	1/8/15	Fukushima	37.735239	140.498299
150203SW2	2/3/15	Soma	37.819648	140.8880746
151020IW	10/20/15	Nihonmatsu	37.535223	140.590019
151021IW	10/21/15	Nihonmatsu	37.540731	140.609687
151022GW	10/22/15	Namie	37.508634	140.931026
151022GW2	10/22/15	Namie	37.479072	140.979392

160607B-1	6/6/16	Namie	37.508634	140.931026
160610B-3	6/10/16	Namie	37.479072	140.979392
160621B-3	6/21/16	Namie	37.510019	140.935489
160719B-1	7/19/16	Namie	37.465361	140.943151
160720B-1	7/20/16	Fukushima	37.761529	140.501009
160720B-2	7/20/16	Fukushima	37.761529	140.501009
160720B-3	7/20/16	Fukushima	37.761529	140.501009
160720B-4	7/20/16	Fukushima	37.761529	140.501009
160720B-5	7/20/16	Fukushima	37.761529	140.501009
160720B-8	7/20/16	Fukushima	37.761529	140.501009
160720B-9	7/20/16	Fukushima	37.761529	140.501009
160720B-10	7/20/16	Fukushima	37.761529	140.501009
160720B-11	7/20/16	Fukushima	37.761529	140.501009
160722B-1	7/22/16	Namie	37.5052778	140.925
160720B-7	7/20/16	Fukushima	37.761529	140.501009
160919B-2	9/19/16	Nihonmatsu	37.542977	140.612194
161025B	10/25/18	Okuma	37.443004	141.007252
161101B-1	11/1/16	Namie	37.507298	140.932306
161101B-3	11/1/16	Namie	37.478359	140.977474
161101B-3	11/1/16	Namie	37.478359	140.977474
161111B-2	11/11/16	Namie	37.465529	140.947112
161111B-3	11/11/16	Namie	37.465529	140.947112
161111B-4	11/11/16	Namie	37.465529	140.947112
161111B-6	11/11/16	Namie	37.465529	140.947112
160917B-1	9/17/16	Nihonmatsu	37.47259	140.648111
161025O-174	10/25/16	Okuma	37.414074	140.987373
161026O-175	10/26/16	Okuma	37.393047	140.997004
161026O-176	10/26/16	Okuma	37.393047	140.997004
161026O-177	10/26/16	Okuma	37.393047	140.997004
161117O-198	11/17/16	Okuma	37.408544	140.975109
20161126F-109	11/26/16	Futaba	37.470056	140.994922
20161128O-206	11/28/16	Okuma	37.399016	140.971983
161130-2	11/30/16	Okuma	37.399016	140.971983
161130-3	11/30/16	Okuma	37.384897	141.009965
161130-4	11/30/16	Okuma	37.384897	141.009965
161206O-229	12/6/16	Okuma	37.390051	140.998834
161213O-246	12/13/16	Okuma	37.384897	141.009965
161213PF26	12/13/16	Futaba	37.459737	141.029345
O-150	10/12/16	Okuma	37.400028	140.989244
TS-24	11/28/16	Namie	37.56192	140.746675
161216BO-247	12/16/16	Okuma	37.414776	140.944361
161208O-237	12/8/16	Okuma	37.390051	140.998834
161208O-239	12/8/16	Okuma	37.408544	140.975109
161208O-240	12/8/16	Okuma	37.408544	140.975109
161208O-242	12/8/16	Okuma	37.384897	141.009965
170113TB-1	1/13/17	Tochigi	36.448364	139.649656
170113TB-2	1/13/17	Tochigi	36.448364	139.649656
170121B-1 O-266	1/21/17	Okuma	37.384897	141.009965

170121B-2 O-267	1/21/17	Okuma	37.384897	141.009965
170926B-4	9/26/17	Okuma	37.38489	141.01008
170926B F-103	9/26/17	Futaba	37.44469	140.987717
170926B F-104	9/26/17	Futaba	37.44469	140.987717
170926B F-105	9/26/17	Futaba	37.44469	140.987717
170926B F-106	9/26/17	Futaba	37.44469	140.987717
170926B T-21	9/26/17	Tomioka	37.340597	140.973536
170926B T-22	9/26/17	Tomioka	37.340597	140.973536
170927B-1	9/27/17	Futaba	37.43066	140.99888
170928B-5 F108	9/28/17	Futaba	37.44469	140.987717
170928B-4 F109	9/28/17	Futaba	37.44469	140.987717
170928B-6 F110	9/28/17	Futaba	37.44469	140.987717
170928B-3 O-110	9/28/17	Okuma	37.402721	140.931879
170928B-2 O-111	9/28/17	Okuma	37.402721	140.931879
170928B-1 O-112	9/28/17	Okuma	37.402721	140.931879
170929B-1 F-111	9/28/17	Futaba	37.44469	140.987717
Wednesday, September 26, 2007	9/26/07	Miyagi	38.20607	140.657342
Friday, August 31, 2007	8/31/07	Miyagi	37.980507	140.829663
Wednesday, September 26, 2007	9/26/07	Miyagi	37.942444	140.773731
Sunday, September 16, 2007	9/16/07	Miyagi	37.917442	140.821556
Tuesday, October 30, 2007	10/30/07	Miyagi	37.97902	140.827388
Saturday, October 13, 2007	10/13/07	Miyagi	37.860541	140.834759
Monday, October 1, 2007	10/1/07	Miyagi	37.85977	140.727458
Wednesday, October 3, 2007	10/3/07	Miyagi	37.862087	140.837674
Saturday, October 20, 2007	10/20/07	Miyagi	38.002148	140.657038
Sunday, November 25, 2007	11/25/07	Miyagi	37.940441	140.861311
Wednesday, November 21, 2007	11/21/07	Miyagi	38.034768,	140.724832
Friday, November 23, 2007	11/23/07	Miyagi	37.936217	140.67745
Wednesday, November 21, 2007	11/21/07	Miyagi	37.919597	140.643785
Wednesday, January 30, 2008	1/30/08	Miyagi	38.231303	140.703927
Saturday, February 2, 2008	2/2/08	Miyagi	38.231303	140.703927
Sunday, December 9, 2007	12/9/07	Miyagi	37.942231	140.868265
Friday, February 8, 2008	2/8/08	Miyagi	38.068945	140.594536
Thursday, February 21, 2008	2/21/08	Miyagi	37.919089	140.588065
Thursday, October 9, 2008	10/9/08	Miyagi	38.232918	140.716632
Thursday, August 14, 2008	8/14/08	Miyagi	37.919256	140.647861
Friday, September 12, 2008	9/12/08	Miyagi	37.931974	140.689212
Tuesday, September 16, 2008	9/16/08	Miyagi	37.876862	140.76097
Tuesday, September 9, 2008	9/9/08	Miyagi	37.905818	140.679528
Monday, September 8, 2008	9/8/08	Miyagi	37.924608	140.780611
Tuesday, September 9, 2008	9/9/08	Miyagi	38.01441	140.640384
Sunday, September 7, 2008	9/7/08	Miyagi	38.01441	140.640384
Friday, August 29, 2008	8/29/08	Miyagi	37.983619	140.831123
Sunday, August 24, 2008	8/24/08	Miyagi	38.02183	140.709601
Tuesday, June 24, 2008	6/24/08	Miyagi	38.128646	140.652416
Sunday, August 31, 2008	8/31/08	Miyagi	38.059892	140.583715
Tuesday, March 16, 2010	3/16/10	Miyagi	38.121091	140.742262
Friday, August 27, 2010	8/27/10	Miyagi	38.035212	140.705883

Friday, January 2, 2009	1/2/09	Yamagata	38.323177	140.383509
Wednesday, July 30, 2008	7/30/08	Yamagata	38.037899	140.25432
Sunday, January 29, 2006	1/29/06	Yamagata	38.545122	140.427827
Tuesday, July 22, 2008	7/22/08	Fukushima	37.409604	139.871889
Wednesday, March 19, 2008	3/19/08	Fukushima	37.2807	140.470144
Wednesday, August 27, 2008	8/27/08	Fukushima	37.795945	140.878681
180526-C1	5/26/18	Nihonmatsu	37.58291	140.59495
180526-C2	5/26/18	Nihonmatsu	37.58567	140.59799
180526-C3	5/26/18	Nihonmatsu	37.64065	140.54304
180528-C1	5/28/18	Nihonmatsu	37.62552	140.56400
180528-C2	5/28/18	Nihonmatsu	37.61360	140.58242
180601-C1	6/1/18	Nihonmatsu	37.60494	140.60372
180602-C1	6/2/18	Nihonmatsu	37.56360	140.63313
180604-C1	6/4/18	Nihonmatsu	37.61154	140.59276
180604-C2	6/4/18	Nihonmatsu	37.64141	140.55642
180607-N1	6/7/18	Namie	37.47842	140.98401
180612-C1	6/12/18	Nihonmatsu	37.58072	140.68183
180614-N1	6/14/18	Namie	37.47010	140.97720
180618-N1	6/18/18	Namie	37.46536	140.92337
180618-N2	6/18/18	Namie	37.47069	140.93422
180618-N3	6/18/18	Namie	37.47846	140.98405
180625-N1	6/25/18	Namie	37.46273	140.92229
180625-N2	6/25/18	Namie	37.46539	140.92339
180625-N3	6/25/18	Namie	37.47285	140.93721
180626-N1	6/26/18	Namie	37.47404	140.96748
180627-C1	6/27/18	Nihonmatsu	37.58633	140.59727
180627-C2	6/28/18	Nihonmatsu	37.58599	140.59798
180629-N1	6/29/18	Namie	37.46273	140.92229
180629-N2	6/29/18	Namie	37.46983	140.97787
180629-N3	6/29/18	Namie	37.47069	140.93422
180629-N4	6/29/18	Namie	37.47434	140.94080
180702-O1	7/2/18	Okuma	37.39314	140.99727
180703-O1	7/3/18	Okuma	37.40044	140.98030
180703-O2	7/3/18	Futaba	37.42617	140.97383
180704-O1	7/4/18	Okuma	37.39168	141.02210
180704-O2	7/4/18	Futaba	37.45594	141.00273
180704-O2_P1	7/4/18	Futaba	37.45594	141.00273
180704-O2_P2	7/4/18	Futaba	37.45594	141.00273
180704-O2_P3	7/4/18	Futaba	37.45594	141.00273
180704-O2_P4	7/4/18	Futaba	37.45594	141.00273
180706-N1	7/6/18	Namie	37.49091	140.93294
180706-N2	7/6/18	Namie	37.48486	140.94446
180707-O1	7/7/18	Okuma	37.40005	140.98935
180709-N1	7/9/18	Namie	37.51989	140.81693
180709-N1_P1	7/9/18	Namie	37.51989	140.81693
180709-N1_P2	7/9/18	Namie	37.51989	140.81693
180709-N1_P3	7/9/18	Namie	37.51989	140.81693
181004-O2	10/4/18	Futaba	37.44757	141.00696

181005-O1	10/5/18	Nihonmatsu	37.33038	141.23210
181006-O4	10/6/18	Nihonmatsu	37.32406	141.24639
181006-O2	10/6/18	Nihonmatsu	37.31774	141.26068
181017-O1	10/17/18	Okuma	37.39104	140.97245
181018-O1	10/18/18	Futaba	37.45054	140.97338
181018-O3	10/18/18	Futaba	37.44852	141.01147
181018-O4	10/18/18	Futaba	37.45240	141.01188
181018-O5	10/18/18	Futaba	37.44852	141.01147
181018-O6	10/18/18	Okuma	37.40854	140.97512
181018-O7	10/18/18	Okuma	37.41645	140.98881
181019-O1	10/19/18	Okuma	37.40588	140.99158
181020-O1	10/20/18	Okuma	37.44751	141.00710
181022-O1	10/22/18	Okuma	37.40588	140.99158
181022-O2	10/22/18	Okuma	37.39788	140.99403
181022-O3	10/22/18	Futaba	37.45240	141.00617
181022-O4	10/22/18	Futaba	37.44412	140.99710
181023-O1	10/22/18	Tomioka	37.37040	141.03414
181024-O1	10/24/18	Okuma	37.41354	140.98786
181024-O2	10/24/18	Okuma	37.40601	140.98786
181024-O3	10/24/18	Okuma	37.40649	140.97299
181024-O4	10/24/18	Futaba	37.46784	140.98933
181025-O1	10/25/18	Okuma	37.40588	140.99158
181027-O1	10/27/18	Futaba	37.44306	140.99483
181029-O1	10/29/18	Tomioka	37.36706	141.03057
181029-O2	10/29/18	Tomioka	37.36706	141.03057
181029-O3	10/29/18	Okuma	37.40562	140.99069
181029-O4	10/29/18	Okuma	37.38414	141.00950
181029-O5	10/29/18	Okuma	37.38785	140.99272
181030-O1	10/30/18	Okuma	37.42089	141.01190
181030-O2	10/30/18	Okuma	37.38769	140.99859
181030-O3	10/30/18	Tomioka	37.35632	141.00368
181031-O1	10/31/18	Okuma	37.40533	140.98400
181031-O2	10/31/18	Tomioka	37.36355	141.00608
181101-O1	11/1/18	Okuma	37.42082	141.01201
181105-O1	11/5/18	Futaba	37.43066	140.99888
181105-O2	11/5/18	Futaba	37.43066	140.99888
181105-O3	11/5/18	Futaba	37.43066	140.99888
181105-O4	11/5/18	Futaba	37.46069	140.99268
181105-O5	11/5/18	Futaba	37.46069	140.99268
181105-O6	11/5/18	Okuma	37.39421	141.00413
181106-O1	11/6/18	Okuma	37.41636	140.99569
181106-O2	11/6/18	Futaba	37.43019	140.98796
181107-O1	11/7/18	Futaba	37.46403	140.97331
181107-O2	11/7/18	Futaba	37.46403	140.97331
181108-O1	11/8/18	Katsurao	37.51463	140.83063
181108-O2	11/8/18	Futaba	37.46012	141.01546
181108-O3	11/8/18	Tomioka	37.36992	141.00054
181109-O1	11/9/18	Okuma	37.39603	141.00072

181109-O1	11/9/18	Tomioka	37.36992	141.00054
181112-O1	11/12/18	Okuma	37.41636	140.995689
181112-O2	11/12/18	Okuma	37.39763	140.972983
181113-O1	11/13/18	Okuma	37.40588	140.99158
181113-O2	11/13/18	Okuma	37.38889	141.00410
181113-O3	11/13/18	Futaba	37.44409	141.00461
181113-O4	11/13/18	Namie	37.48155	140.94243
181114-O1	11/14/18	Okuma	37.38889	141.00410
181114-O2	11/14/18	Okuma	37.38889	141.00410
181114-O3	11/14/18	Okuma	37.38117	141.01386
181115-O1	11/15/18	Tomioka	37.36409	141.00991
181115-O2	11/15/18	Tomioka	37.36498	141.00980
181115-O3	11/15/18	Futaba	37.45821	141.00376
181116-O1	11/16/18	Namie	37.46644	140.94704
181117-O1	11/17/18	Futaba	37.46263	141.00484
181117-O2	11/17/18	Futaba	37.46263	141.00484
181117-O3	11/17/18	Futaba	37.43066	140.99888
181117-O4	11/17/18	Namie	37.46574	140.95640
181117-O5	11/17/18	Namie	37.50506	140.94531
181119-O1	11/19/18	Tomioka	37.36409	141.00991
181119-O2	11/19/18	Okuma	37.40838	140.95689
181119-O3	11/19/18	Futaba	37.46464	140.99466
181119-O4	11/19/18	Futaba	37.43066	140.99888
181119-O5	11/19/18	Futaba	37.44871	141.01962
181119-O6	11/19/18	Futaba	37.43066	140.99888
181121-O1	11/21/18	Katsurao	37.5229	140.8133
181121-O2	11/21/18	Katsurao	37.51874	140.82133
181121-O3	11/21/18	Okuma	37.39421	141.00413
181121-O4	11/21/18	Okuma	37.40396	140.98682
181122-O1	11/22/18	Namie	37.48920	140.94244
181122-O2	11/22/18	Futaba	37.46403	140.97331
181124-O1	11/24/18	Futaba	37.44361	140.98507
181124-O2	11/24/18	Futaba	37.44361	140.98507
181124-O3	11/24/18	Okuma	37.41600	140.99514
181126-O1	11/26/18	Namie	37.46574	140.95640
181126-O2	11/26/18	Namie	37.45831	140.94283
181126-O3	11/26/18	Namie	37.45831	140.94283
181126-O4	11/26/18	Tomioka	37.35626	141.02689
181126-O5	11/26/18	Namie	37.48155	140.94243
181127-O1	11/27/18	Okuma	37.38258	141.00663
181127-O2	11/27/18	Okuma	37.38258	141.00663
181127-O3	11/27/18	Okuma	37.40356	140.98143
181127-O4	11/27/18	Tomioka	37.36506	141.02599
181128-O1	11/28/18	Okuma	37.39689	140.98842
Tuesday, October 30, 2007	10/30/07	Miyagi	37.984304	140.831674
Sunday, November 18, 2007	11/18/07	Miyagi	37.983938	140.762819
Thursday, February 14, 2008	2/14/08	Miyagi	38.353915	140.678179
F1	12/15/07	Fukushima	37.53221	140.556874

S2	2/7/11	Yamagata	38.054387	140.146612
y1	12/27/07	Yamagata	38.124723	140.293803
soumaI	5/8/08	Fukushima	37.734793	140.638163
140528B	5/28/14	Fukushima	37.749246	140.4729678
161022OT-33	10/22/16	Okuma	37.388383	140.968505
180626-N3	6/26/18	Namie	37.4720652	140.9423861
181017-O1	10/17/18	Okuma	37.39104	140.97245
181018-O2	10/18/18	Futaba	37.43622	140.97741
